# Shift Toward Randomness in Brain Networks of Patients With Anorexia Nervosa: The Role of Malnutrition

**DOI:** 10.3389/fnins.2021.645139

**Published:** 2021-03-24

**Authors:** Enrico Collantoni, Paolo Meneguzzo, Elena Tenconi, Valentina Meregalli, Renzo Manara, Angela Favaro

**Affiliations:** ^1^Department of Neurosciences, University of Padua, Padua, Italy; ^2^Padova Neuroscience Center, University of Padua, Padua, Italy

**Keywords:** eating disorders, anorexia nervosa, diffusion tensor imaging – fiber tractography, graph theory, brain networks, small-world architecture

## Abstract

No study to date investigated structural white matter (WM) connectome characteristics in patients with anorexia nervosa (AN). Previous research in AN found evidence of imbalances in global and regional connectomic brain architecture and highlighted a role of malnutrition in determining structural brain changes. The aim of our study was to explore the characteristics of the WM network architecture in a sample of patients with AN. Thirty-six patients with AN and 36 healthy women underwent magnetic resonance imaging to obtain a high-resolution three-dimensional T1-weighted anatomical image and a diffusion tensor imaging scan. Probabilistic tractography data were extracted and analyzed in their network properties through graph theory tools. In comparison to healthy women, patients with AN showed lower global network segregation (normalized clustering: *p* = 0.029), an imbalance between global network integration and segregation (i.e., lower small-worldness: *p* = 0.031), and the loss of some of the most integrative and influential hubs. Both clustering and small-worldness correlated with the lowest lifetime body mass index. A significant relationship was found between the average regional loss of cortical volume and changes in network properties of brain nodes: the more the difference in the cortical volume of brain areas, the more the increase in the centrality of corresponding nodes in the whole brain, and the decrease in clustering and efficiency of the nodes of parietal cortex. Our findings showed an unbalanced connectome wiring in AN patients, which seems to be influenced by malnutrition and loss of cortical volume. The role of this rearrangement in the maintenance and prognosis of AN and its reversibility with clinical improvement needs to be established by future studies.

## Introduction

The neurobiological characterization of anorexia nervosa (AN) through structural and functional neuroimaging techniques suggests that the complex array of symptoms characterizing AN emerges from failures in the relations between multiple areas rather than from distinct regional alterations (Frank, 2014; Steward et al., 2017). The brain networks that were found to be altered in AN are functionally and structurally supported by spatially distributed neurocircuits and are involved in different processes, which include cognitive control, reward processing, self-monitoring, visuospatial and somatosensory functions, emotion recognition, and social/interpersonal abilities (Favaro et al., 2012; Ehrlich et al., 2015; Collantoni et al., 2016; Frank et al., 2016; Seidel et al., 2018). The presence of alterations in the connectivity between topologically distributed areas has suggested that AN is characterized by imbalances in the properties that regulate their functional and structural integration. In recent years, graph theory tools have been successfully used to investigate the rules that govern the interrelations between distinct brain areas and to explore their influence on specific psychological, cognitive, and behavioral traits (Bullmore and Sporns, 2009). Moreover, the application of graph theory to neuroimaging data allows describing the organizational properties of neural networks. From a global perspective, the organization of a neural network can be inferred by parameters that can describe its ability to efficiently integrate the communication between distant brain regions, such as global efficiency and characteristic path length and by indices that can explain its capacity to ensure adequate processing of local information, such as modularity and clustering coefficient. On a regional level, one of the most relevant contributions of connectomic analysis is its ability to identify the most influential and connected regions within the brain. These regions are called hubs and were shown to be particularly vulnerable and sensitive to pathogenic mechanisms affecting the brain and are also likely to spread disorder-related processes to other brain areas (Rubinov and Sporns, 2010; Fornito et al., 2017).

Connectomic approaches have been recently used with both functional and anatomical data in different psychiatric disorders such as schizophrenia, major depressive disorder, obsessive–compulsive disorder, and attention-deficit/hyperactivity disorder (Rubinov and Bullmore, 2013; Taylor, 2014; Sidlauskaite et al., 2015). Studies that evaluated network architecture in AN have been focused on the covariance patterns between cortical structural data and functional connectivity measures, revealing the presence of imbalances in segregation and integration properties of brain connectome (Geisler et al., 2018; Collantoni et al., 2019a, b). Interestingly, the studies evaluating cortical morphological and functional connectivity patterns did not report consistent findings in terms of segregation and integration. Indeed, the observations conducted on cortical structural indices reported higher levels of segregation among clusters, whereas functional data showed a less efficient transfer of information, due to a reduced efficiency and clustering of the connectome. Overall, these results suggest that the alterations of these networks may underpin distinct pathophysiological processes, which are likely to impact differently on cortical morphological and on functional connectivity patterns. Therefore, although no study has directly evaluated the white matter (WM) structural connectome to date, this analysis could be particularly useful for combining structural information with a more direct analysis of connectivity patterns in the brain. Moreover, a connectomic evaluation of WM in AN would allow to explore the effects of starvation on brain connectivity from both a global and a regional point of view. The onset of AN occurs in adolescence when WM development typically reflects the need to recruit prefrontal areas in cognitive control/top-down abilities and the demand for association and projection fibers that support the integration of cortical and subcortical processes (Asato et al., 2010). Interestingly, imbalances in the integration between top-down frontoparietal networks and subcortical limbic ones have recently been proposed to be central in the neurobiology of AN, probably as the result of alterations in the maturation of cortical areas or as a consequence of malnutrition processes (Favaro, 2013; O’Hara et al., 2015; Collantoni et al., 2016). The analysis of the global and regional properties of WM patterns could be meaningful from this point of view as its architecture is fundamental to guarantee an adequate flow of information across the brain and to integrate signals from topologically separated areas. Moreover, the possibility to detect any alterations in brain connectomic measures could help to better understand the pathogenetic processes underlying specific psychopathological, temperamental, and cognitive traits characterizing the disorder and therefore to identify brain-directed treatment strategies (Fox et al., 2014).

The aim of the present research is to analyze WM architecture employing graph theory tools in a sample of patients with acute AN, to test group differences in hub distribution, and to observe the presence of any correlation between clinical variables (including weight loss) and the properties of the graph. We hypothesized a global rearrangement of network properties as a form of adaptation of brain functioning to the effects of malnutrition to maintain integration and efficiency. We also hypothesized an alteration of segregation and centrality properties of brain nodes in areas (parietal and limbic cortex) suspected to be involved in the core psychopathology of AN.

## Materials and Methods

A total of 36 patients with acute AN and 36 healthy women (HW) were included in this study (age range, 15.54–40.52 years). Participants of the present study also participated in previous studies of our group (Favaro et al., 2012; Collantoni et al., 2016, 2017). Exclusion criteria for both the AN and the HW groups were male gender, pregnancy, active use of systemic steroids, bipolar disorder or schizophrenia spectrum disorder, major depression, active suicidality, moderate mental impairment (IQ < 60) or learning disabilities, history of substance/alcohol abuse or dependence, use of medications other than antidepressants, history of any serious neurological or medical illness, history of head trauma or injury with loss of consciousness, and known contraindications to conventional magnetic resonance imaging (MRI). For HW, additional exclusion criteria were a history of any psychiatric disorder and the presence of any first-degree relatives with an eating disorder. The main clinical characteristics of the sample are reported in Table 1.

**TABLE 1:** Main clinical characteristics of patients with AN and healthy women.

	AN patients (**n** = 36)	Healthy women (**n** = 36)	AN vs. HW
			*z*(p)
			
	Mean(SD)	Mean (SD)	
Age (years)	26.3(7.3)	25.4 (6.4)	0.25 (0.804)
Baseline BMI (kg/m^2^)	15.9(1.8)	21.7 (3.0)	7.21 (0.000)
Lowest BMI (kg/m^2^)	14.0(1.8)	19.8 (2.5)	6.95 (0.000)
Age at onset (years)	18.4(5.2)	–	–
Duration of illness (months)	79.7(83.3)	–	–
Edinburgh laterality index	56.2(38.4)	54.5 (43.0)	0.46 (0.648)
Education (years)	14.1(2.2)	15.5 (2.3)	2.87 (0.004)
Drive for thinness*	10.1(6.0)	2.4 (4.2)	5.41 (0.000)
Depression*	1.3(0.7)	0.8 (0.6)	3.38 (0.001)
Trait anxiety*	56.2(9.8)	39.9 (9.5)	5.56 (0.000)
Block Design Unsegmented	31.2(9.3)	37.9 (7.5)	2.91 (0.004)
Brief Intelligence Test	104.6(5.5)	107.2 (3.0)	2.52 (0.012)

*Drive for thinness was evaluated using the Eating Disorders Inventory; depression was evaluated using the Hopkins Symptoms Checklist; and anxiety was evaluate using the State-Trait Anxiety Inventory. Block Design Unsegmented is part of the revised version of the Wechsler Adult Intelligence Scale.

When recruiting subjects, some of them were not included in the study: five AN patients with AN, because of antipsychotic medication and/or severe comorbidity; one AN patient and one healthy subject, because of previous head trauma; and one patient with AN and two healthy subjects, who were not available to undergo MRI scanning when scheduled. Finally, four other participants (two AN patients and two healthy women) were excluded for technical reasons (DTI module not available on the day of scanning). The final sample comprised 72 women (36 with AN and 36 HW). No further subject was excluded because of problems with scan acquisition, motion artifacts, or gross brain alterations.

All experiments were performed in accordance with relevant named guidelines and regulations. Ethical permission was obtained from the Ethics Committee of the University Hospital of Padua. After completely describing the study to the subjects, informed written consent was obtained.

### Clinical Assessment

All subjects were investigated for AN diagnosis using a diagnostic interview according to the Eating Disorders Section of the Structured Clinical Interview for *Diagnostic and Statistical Manual of Mental Disorders, Fifth Edition* (*DSM-5*) (American Psychiatric Association [APA], 2013). A semistructured interview was also performed to collect sociodemographic and clinical variables (Favaro et al., 2013). All the subjects completed the Hopkins Symptoms Checklist (Derogatis et al., 1974) to assess depressive symptoms, the State-Trait Anxiety Inventory (Spielberger et al., 1999), and the Eating Disorders Inventory-1 (Garner et al., 1983) to assess eating psychopathology. The Edinburgh Handedness Inventory (Oldfield, 1971) was used to assess handedness. Visuospatial and verbal abilities were measured, respectively, by the Unsegmented Block Design subtest of the revised version of the Wechsler Adult Intelligence Scale (Wechsler, 1981) and the Brief Intelligence Test (the Italian version of the National Adult Reading Test) (Colombo et al., 2002). Measures of verbal and visuospatial abilities were significantly intercorrelated in healthy women (ρ = 0.51, *p* = 0.01), but not in patients (ρ = 0.06, *p* = 0.802). All subjects were recruited at the University Hospital of Padua Eating Disorders Unit, and all fulfilled the *DSM-5* criteria for AN. At the time of scanning, all of them were medically stable. The diagnostic subtype at the time of scanning was as follows: restrictive in 32 patients (84%) and binge-eating/purging type in six patients. Seven patients who were restricting at the time of the present study reported previous recurrent binge eating and/or purging. Concerning the use of medications, 14 AN patients were under treatment with antidepressant agents at the time the study was conducted (acute AN: one case mirtazapine, two paroxetine, two escitalopram, one fluoxetine, eight sertraline).

### MRI Data Acquisition

Data were collected on a Philips Achieva 1.5-T scanner equipped for echo-planar imaging. A high-resolution three-dimensional T1-weighted anatomical image was also acquired, in a gradient-echo sequence (repetition time = 20 s, echo time = 3.78 ms, flip angle = 20°, 160 sagittal slices, acquisition voxel size = 1 mm × 0.66 mm × 0.66 mm, field of view 21–22 cm).

### Image Processing

All DICOM images were converted to NIfTI format. Diffusion gradients were extracted using mriconvert^1^. The package FMRIB Software Library’s (FSL’s) Diffusion Toolkit was used to preprocess diffusion-weighted images and for the diffusion tensor estimation.

### Connectivity Matrices

Each subject’s brain was parcelated into 162 regions of interest (ROIs): 148 cortical regions of the Destrieux atlas and 14 subcortical regions, all obtained by automated extraction tools (Freesurfer)^2^. The quality of the parcelation was manually checked for each subject.

The structural connectivity between the brain regions was investigated with probabilistic tractography with the Brain Diffusion Toolbox (version FSL 4.1.6^3^; FMRIB, Oxford, United Kingdom) (Behrens et al., 2007). Pathways were tracked with any parcelated brain region as seed and all the other 161 ROIs as targets. Connectivity between ROIs was defined as the number of probabilistic streamlines arriving in one ROI when another ROI was seeded and *vice versa*. Seeding and streamline counting was performed in the voxels within the ROI that were in the gray matter/WM boundary. We used the default parameters of two fibers per voxel and 5,000 sample streamlines for each tract to create a 148 × 148 matrix, *P*, of probability values. Each matrix entry Pij represents a scaled conditional probability of a pathway between the seed ROI, *i*, and the target ROI, *j*, given by *Pij* = (*Si*→*j*/*Si*) *Ri*, where *Si*→*j* denotes the number of the fibers that reach the target region *j* from the seed region *I*, and *Si* is the number of streamlines seeded in *i*. This measure quantifies connectivity such that *Pji* ≈ *Pji*, which, on averaging, gives an undirected weighted connectivity measure. This now creates a 162 × 162 undirected symmetrical weighted connectivity network.

### Network Properties and Statistical Analyses

Graph network properties of the connectome were computed using integration, segregation, and centrality indices. Integration was measured using characteristic path length and global efficiency; segregation was measured using clustering coefficient, local efficiency, modularity, and transitivity. Centrality properties of network nodes were measured using degree and betweenness values. We also quantified the Small-World Index, a measure of the balance between integration and segregation. The Small-World Index is computed as the ratio between two key metrics: the normalized clustering coefficient and the normalized characteristic path length of the network (for a more detailed description of the parameters, see the Supplementary Material).

The network measures were computed using Graph Analysis Toolbox^4^ (Hosseini et al., 2012). The effects of age and Edinburgh Handedness Inventory score were removed by applying a linear regression analysis to each regional morphometry data. The residuals of these regressions were used for the analyses. A non-parametric permutation test with 1,000 repetitions was used to search for differences in topological indices. The numerosity of the original groups was maintained in each repetition by randomly reassigning regional data residuals of each participant to one of the two groups, to obtain an association matrix for each random group. A range threshold of 0.02–0.2 with increments of 0.02 was then applied to each random group to calculate the binary adjacency matrices.

For all networks, topological measurements were estimated, and the full density range was used to compare differences. The values of each group across the range of density were plotted, and differences of areas under the curve (AUCs) were used to compute the topological properties for each iteration. Probability (*p*) values were computed by comparing the results from the actual differences in the curve functions and the null distribution of differences. This non-parametric permutation test compared the shapes of the curves derived from multiple threshold points and is based on functional data analysis (FDA).

The same permutation procedure used to test the significant between-group differences at a whole-brain level was used to compare regional indices. The significance of regional comparisons was corrected using the false discovery rate method. The nodes whose FDA-based curve functions for regional degree and betweenness were 2 standard deviations higher than the mean of corresponding curve functions computed from the 1,000 random permutations were considered as Hubs.

The topological properties of the single nodes of the same lobe were averaged and compared between groups (Supplementary Table 1). The differences between patients and healthy women in the cortical volumes of the parcelated regions were averaged for any lobe, and their relationships with averaged topological properties of the same lobe were tested by linear regression analysis. The Mann–Whitney *U* test was applied for the group comparison of the main clinical variables. Correlations between clinical variables (those included in Table 1), and topological properties were tested using Spearmen ρ (significant level of *p* < 0.05, uncorrected).

## Results

The main findings regarding the properties of structural networks and the distribution of the nodes with the highest betweenness and degree (hubs) are reported in Table 2.

**TABLE 2:** Network properties and hub distribution in patients with AN and healthy women.

	Patients with AN (**n** = 36)	Healthy women (**n** = 36)	AUC
			p
			
	Global measures mean (SD)	Mean (SD)	
Small-World Index	3.97 (0.22)	4.08 (0.20)	0.031
Normalized clustering coefficient	5.34 (0.34)	5.50 (0.30)	0.029
Mean local efficiency	0.71 (0.01)	0.71 (0.00)	0.334
Transitivity	0.42 (0.01)	0.43 (0.01)	0.054
Modularity	0.52 (0.01)	0.52 (0.01)	0.791
Global efficiency	0.43 (0.00)	0.43 (0.00)	0.462
Characteristic path length	3.09 (0.06)	3.10 (0.06)	0.626

	**Regional measures mean (SD)**	**Mean (SD)**	

Right anterior cingulate gyrus clustering coefficient	1.03 (0.11)	0.93 (0.11)	0.000
Right fusiform gyrus betweenness	0.23 (0.10)	0.16 (0.08)	0.000
Net hub degree	Left pericallosal sulcus	Left superior frontal gyrus	
	Left putamen	Left pericallosal sulcus	
	Left thalamus	Left putamen	
	Right superior frontal gyrus	Left thalamus	
	Right pericallosal sulcus	Right superior frontal gyrus	
	Right putamen	Right pericallosal sulcus	
		Right putamen	
Net hub betweenness	Left superior frontal gyrus	Left superior frontal gyrus	
	Left pericallosal sulcus	Left superior parietal lobule	
	Left putamen	Left pericallosal sulcus	
	Left thalamus	Left putamen	
	Right superior frontal gyrus	Left thalamus	
	Right pericallosal sulcus	Right superior frontal gyrus	
	Right putamen	Right superior occipital gyrus	
		Right pericallosal sulcus	
		Right putamen	

On a global level, patients with AN showed a reduced Small-World Index (*p* = 0.031) and a lower normalized clustering coefficient (*p* = 0.029). Transitivity, which estimates the relative number of triangles in the graph, compared to the total number of connected triples of nodes, shows a tendency toward a reduction in patients with AN (*p* = 0.054). No differences were observed in other global network measures between patients with AN and healthy women.

In the regional analyses, patients with AN showed a significantly higher clustering coefficient in the right anterior cingulate gyrus (*p* < 0.001) and a higher betweenness in the right fusiform gyrus (*p* < 0.001).

**FIGURE 1:**
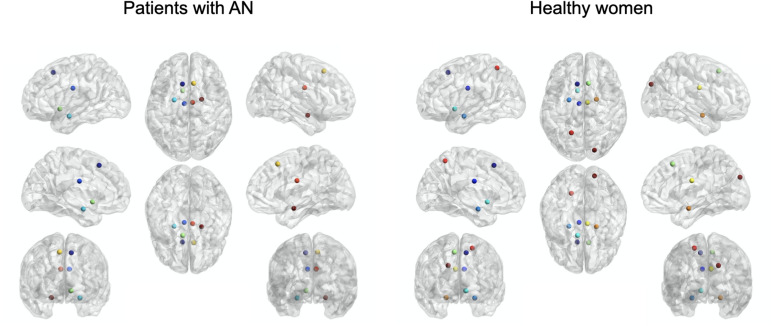
Hub distribution based on betweenness values in patients with AN and healthy women.

Based on the betweenness, we identified the same hub distribution in the two groups, except for the left superior parietal lobule and the right superior occipital gyrus, which were present only in healthy women and not in patients with AN (Figure 1).

Based on the degree, we identified an identical hub distribution between patients and controls, except for the right superior frontal gyrus, which was lacking in the patient group (Supplementary Figure 1).

On a global level, in the experimental group, significant positive correlations emerged between the Small-World Index and both the lifetime lowest body mass index (BMI) (ρ = 0.568, *p* < 0.001) and the BMI at the time of scanning (ρ = 0.376, *p* = 0.024) and between the normalized clustering coefficient and the lifetime lowest BMI (ρ = 0.476, *p* = 0.027). Correlations between regional topological variables that showed significant between-group differences and clinical variables were also explored. The clustering coefficient of the right anterior cingulate cortex (ACC) significantly correlated with visuospatial abilities in the AN group (ρ = 0.42, *p* = 0.015) and with the BMI at scanning (ρ = −0.382, *p* = 0.022) in healthy women. The betweenness of the right fusiform gyrus significantly correlated with visuospatial abilities (ρ = 0.40, *p* = 0.021) in healthy women only.

**FIGURE 2:**
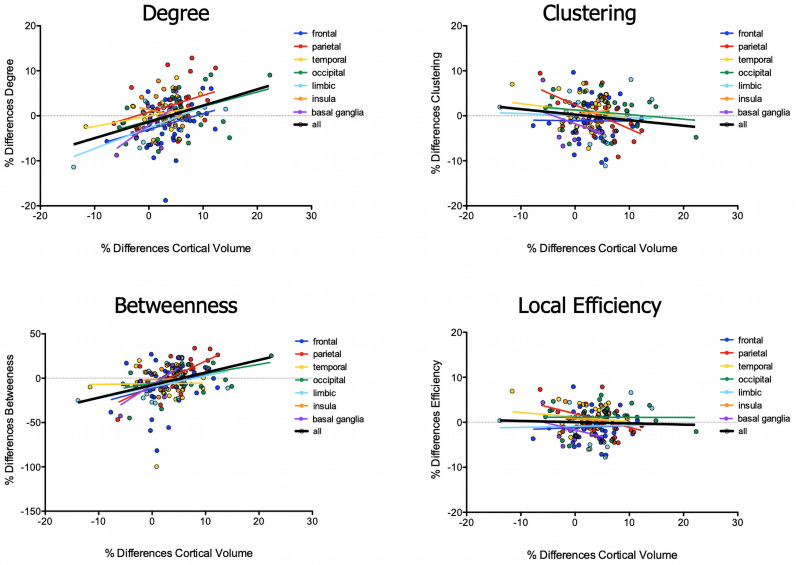
Correlation between differences in brain volumes and topography values of brain nodes.

Exploring the relationship between differences in volumes of brain areas and changes in local topological characteristics of the corresponding nodes, we found (1) increased values of degree along with increased rates of volumetric differences between patients and healthy women in occipital (*p* = 0.0188, *R*^2^ = 0.194), limbic (*p* = 0.0025, *R*^2^ = 0.490), basal ganglia (*p* = 0.0044, *R*^2^ = 0.505), and overall brain nodes (*p* < 0.001, *R*^2^ = 0.143) (Figure 1); (2) increased values of betweenness along with increased differences in volumes between the two groups in parietal (*p* = 0.0006, *R*^2^ = 0.424), occipital (*p* = 0.031, *R*^2^ = 0.167), basal ganglia (*p* = 0.002, *R*^2^ = 0.565), and overall brain nodes (*p* < 0.0001, *R*^2^ = 0.124 (Figure 1); (3) decreased values of clustering along with increased differences in volume in the parietal lobe (*p* = 0.011, *R*^2^ = 0.259), but not in overall brain nodes (*p* = 0.079, *R*^2^ = 0.019) (Figure 1); and (4) decreased values of local efficiency along with increased differences in volume in nodes of the parietal lobe (*p* = 0.036, *R*^2^ = 0.186), but not in those of the all brain (*p* = 0.66, *R*^2^ = 0.001) (Figure 2).

## Discussion

To our knowledge, the present study is the first one that evaluated WM connectome through graph theory tools in AN. Our data provide evidence of the presence of reduced small-world properties in patients with AN, which seems to be driven by a reduction in global clusterization of the brain networks. The reduction in the segregation characteristics of the connectome in this group does not reflect an increase in network integration as no significant differences emerged between the two groups in integration measures.

As the clusterization of the connectome indicates its tendency to be composed of densely connected and functionally coherent neuronal units (Bullmore and Sporns, 2009), higher clustering levels reflect an increase in the network regularity, whereas lower levels of segregation usually reveal a shift toward more random configurations (Bassett and Bullmore, 2006; Rubinov and Sporns, 2010). Therefore, we can hypothesize that lower Small-World Index values in the AN group, alongside a reduction in the clustering coefficient, could reveal a loss in the regularity of the structural connectivity network in the disorder. The clustering coefficient is also considered an indirect measure of degeneracy (Fornito et al., 2015), which is the capacity of the brain for resilience and compensation. The correlation of this measure with the lowest BMI seems to indicate that the direct consequence of malnutrition is a decrease of the ability of the brain to compensate to a loss of function. The decrease in resilience is more severe in patients who reached very low BMI and showed a relationship with variations in cortical volumes, especially in the parietal cortex. It is noteworthy that a reduced clustering coefficient compared to healthy women has been found in the same sample exploring the functional connectome (Collantoni et al., 2019a) and in an independent sample of patients with AN (Geisler et al., 2018).

This altered balance between integration and segregation properties in patients with AN might be due to disorder-related consequences (i.e., malnutrition), but also derive from preexistent deviations in neurodevelopmental trajectories. The literature about how structural connectivity networks change during brain maturation evidenced processes supporting an interactive specialization between areas. In particular, the connections between unimodal regions are likely to strengthen during childhood, whereas later developmental phases are characterized by increased connections between association areas (Cao et al., 2016; Wierenga et al., 2016). However, longitudinal studies evaluating structural networks development in pathological populations are lacking, and further research is needed. The cross-sectional nature of our data limits any inference about this point. Nevertheless, it seems quite clear that in our sample malnutrition played a significant role, as the lowest lifetime BMI and the current BMI were positively correlated with the small-world properties of the network, and the lowest lifetime BMI was also positively correlated with the clustering coefficient. These results support a role of malnutrition-related processes in determining a loss in network regularity, which is probably due to an alteration in those processes that determine the trade-off between integration and segregation characteristics of the connectome.

The analysis of how differences in network properties of specific brain nodes were associated with differences in cortical volumes of the corresponding brain areas showed specific patterns of relationships in the whole brain, although the magnitude of these association was greater for some brain lobes (in particular, in subcortical, occipital, limbic, and parietal lobes). These results may suggest that cortical volumetric changes might peculiarly impact the structural connectomic architecture in AN, mainly perturbing the nodal centrality. Moreover, our results highlighted that volumetric differences in parietal regions inversely correlated with segregation properties, suggesting a specific sensitivity of parietal regions to volumetric brain alterations. Given that parietal regions participate in many functions that are involved in AN pathophysiology such as in somatosensory integration abilities (Moscovitch and Behrmann, 1994); in the perception of personal, interpersonal, and peripersonal space (Vieira et al., 2019); in social abilities (Parkinson et al., 2014); and in many executive functions (Culham and Kanwisher, 2001), a reduction of their local efficiency and segregation properties together with volumetric changes is particularly interesting and deserves to be further explored. However, giving the cross-sectional nature of our study, we cannot exclude that it is the change in topological properties of brain nodes to influence the loss of cortical volume in the corresponding brain area of patients.

Regional analyses highlighted a significant change in topological network characteristics in two specific brain areas: the right anterior cingulate gyrus, which displays a higher clustering coefficient, and the right fusiform gyrus, which shows higher betweenness values. The ACC is anatomically connected with different cortical, limbic, and paralimbic regions (Margulies et al., 2007) and is functionally involved in several self-referential and self-regulation processes (Carter et al., 1998). In AN, ACC was shown to be involved in the cognitive control of appetite, perfectionism, body image distortion, cognitive inflexibility, and elevated performance monitoring (Friederich et al., 2010; Bischoff-Grethe et al., 2013; Boehm et al., 2014; Lee et al., 2014; Geisler et al., 2017). In our study, the clustering properties of the ACC in healthy women showed a correlation with BMI in the same direction (the lower the weight, the higher the clustering of ACC). In patients, visuospatial abilities were associated with higher clustering in the ACC, providing data in favor of a possible role of ACC in visuoperceptual reasoning in AN with possible implications for body image.

The right fusiform gyrus has been involved in the recognition of familiar faces, visual working memory, and the processing of emotions (Schwarzlose, 2005; Kawasaki et al., 2012). In AN, right fusiform gyrus was found to activate significantly more than in healthy controls during the early processing of facial expressions, suggesting increased functional recruitment of this area probably due to perceptual processing difficulties in this group of patients (Fonville et al., 2014). Our data showed that right fusiform gyrus betweenness correlated with visuospatial abilities in healthy women, but not in patients, thus confirming a possible dysfunction of this brain area in AN. We can also hypothesize that the higher betweenness of the right fusiform gyrus could simply reflect the increase in “traffic” due to the increased functional recruitment of this node.

The architecture of the most central and influential nodes shows some differences between the experimental group and the healthy one. Hubs constitute the architectural backbone of the neural network and facilitate the structural and functional integration of the connectome (Roberts et al., 2018). Several studies reported differences in the distribution of hubs in different psychiatric disorders (Rubinov and Bullmore, 2013), but no research analyzed the hub distribution in AN to date. This seems to be particularly relevant because abnormalities in hub distribution were proposed to be crucial both in the neurobiology of several psychiatric disorders and to design of brain targeted treatment options (Riva-Posse et al., 2014). In patients with AN, we observed a reduction of the number of hubs computed both on the betweenness and the degree values, with a lack of hubs in parietal, occipital, and frontal areas. These areas are involved in several highly integrative functions, which are perceptual, cognitive, and behavioral. A reduction of their centrality in the WM connectivity network could mean that these regions are particularly vulnerable in AN and that they may lose their natural integrative function in the disorder. As betweenness is a measure of the local influence of a node, the decrease of the number of hubs in patients with AN suggests a reorganization of the local information processing in AN and is also consistent with the observation of a more random and less organized WM architecture in the diagnostic group, especially in brain areas that are crucial for perceptual and cognitive processing. It is possible that this reorganization might allow the maintenance of a global network efficiency, at the expense of more problems in cognitive and body-perception functioning.

The present study has both strengths and limitations, which need to be taken into consideration when interpreting its results. It is the first study to analyze the WM connective architecture using graph theory tools in patients with acute AN and to describe the relationships between topological indices and clinical measures. However, the cross-sectional nature of our observations does not allow us to understand whether the observed alterations precede the onset of AN or represent a consequence of the disorder. Moreover, in the computation, we did not control for the potential bias of the partial volume effect and free water due to ventricular enlargement (Kaufmann et al., 2017; Meneguzzo et al., 2019), which, however, are not likely to have a significant effect on the global architecture of the network and on the observed regional alterations.

In conclusion, the present study suggests the presence of a reorganization of the overall WM network in patients with acute AN. The shift toward a more random configuration of the network in AN results in the loss of some of the most integrative and influential hubs in the brain, which are located in parietal and occipital regions. The reorganization of the brain network showed significant relationships with BMI and reduced volumes of gray matter, as an association was evident between differences in topological properties and differences in cortical volumes, particularly in the parietal cortex where lower levels of clusterization and efficiency were observed in the most affected areas. Further studies could be useful to evaluate the impact of weight loss as well as weight loss speed on brain network architecture (Solmi et al., 2018). Moreover, longitudinal evaluations are needed to better understand how much all these alterations are a consequence of malnutrition and how much weight gain is associated with a recovery of network brain organization.

## Data Availability Statement

The raw data supporting the conclusions of this article will be made available by the authors, without undue reservation.

## Ethics Statement

The studies involving human participants were reviewed and approved by the Ethics Committee of the University Hospital of Padua. Written informed consent to participate in this study was provided by the participants’ legal guardian/next of kin.

## Author Contributions

EC, AF, and RM: conceptualization, formal analysis, and methodology. AF and RM: data acquisition. AF: supervision. EC and PM: writing the original draft. AF, ET, and VM: writing, review, and editing. All authors contributed to the article and approved the submitted version.

## Conflict of Interest

The authors declare that the research was conducted in the absence of any commercial or financial relationships that could be construed as a potential conflict of interest.

## Footnotes

1 http://lcni.uoregon.edu/~jolinda/MRIConvert

2 http://surfer.nmr.mgh.harvard.edu

3 http://www.fmrib.ox.ac.uk

4 https://www.nitrc.org/projects/gat/
