# Supplementary material for: Shift Toward Randomness in Brain Networks of Patients With Anorexia Nervosa: The Role of Malnutrition
Source: Front Neurosci. 2021 Mar 24;15:645139. doi: 10.3389/fnins.2021.645139 (PMC8024518; doi:10.3389/fnins.2021.645139)

**Supporting Information**

**Supplementary Methods**

**Graph-based metrics**

Measures of integration, segregation, and centrality were computed on each group-specific graph.

*Integration measures*

1. **Path length** is the minimum number of edges that must be traversed to go from one node to another. It describes the ability of a network to integrate information using shortest path routing. The characteristic path length, which is defined as the mean of geodesic lengths over all couples of nodes, provides a measure of the typical separation between two nodes in the graph.

2. **Global efficiency** is a measure of the information exchange efficiency within a network and is inversely related to path length. It is a measure of the integration of a system, so that a fully connected network has the maximum overall efficiency, while a fully disconnected network has minimal global efficiency values.

*Segregation measures*

1. The **clustering coefficient** can be computed at the level of individual nodes or across the overall network. The clustering coefficient of a node is calculated as the probability that two nodes that are connected with a third node will also be connected, thus describing the density of connections between the neighbors of the node. The clustering coefficient of the global network is computed as the average clustering coefficients across nodes, thus indicating the cliquishness of connectivity in the graph.

2. **Local efficiency** represents the efficiency and the integration of a local area formed by regions that are neighbors of a given node.

3. **Modularity** describes how strongly a network is divided into modules. Modules are densely interconnected subgroups of nodes that are poorly connected with other regions. High rates of connectivity between nodes within a module suggest that they are functionally integrated. Weak connectivity between nodes within different modules indicates a segregation in their functions.

*Centrality measures*

We used two centrality measures to define the hubs in each group connectome: the degree of the nodes and the betweenness centrality.

1. The degree of a node is represented by the number of edges incident with the node.

2. The betweenness centrality of a node measures the proportion of shortest paths between all pairs of nodes in the network that pass through that node.

*Small-world index*

The small world index (SWI) of a structural covariance network indicates the extent to which it is interposed between regular and random graphs. The SWI is computed by comparing the characteristic path length and clustering coefficient of a graph with the corresponding values of null random graphs with the same number of nodes, edges, and degree distribution. A SWI>1 indicates a network that has relatively high segregation and integration compared to random null networks and suggests the presence of an efficient information transfer at a relatively low wiring cost.

**Supplementary Results**

**Supplementary Table 1.** Average topologic values of nodes in the groups

|  | AN patients | Healthy women | AN vs. HW  *z* (p) |
| --- | --- | --- | --- |
|  | (n=36) | (n=36) |  |
|  | Mean (SD) | Mean (SD) |  |
| Clustering: |  |  |  |
| Parietal | 0.927 (0.032) | 0.932 (0.034) | 0.586 (0.558) |
| Frontal | 1.025 (0.020) | 1.019 (0.026) | 0.698 (0.485) |
| Insula | 1.040 (0.049) | 1.048 (0.041) | 0.721 (0.471) |
| Subcortical | 0.881 (0.044) | 0.863 (0.046) | 1.442 (0.149) |
| Occipital | 1.015 (0.038) | 1.020 (0.035) | 0.473 (0.636) |
| Limbic | 1.021 (0.028) | 1.018 (0.046) | 0.146 (0.884) |
| Temporal | 1.044 (0.042) | 1.053 (0.029) | 1.126 (0.260) |
| Degree: |  |  |  |
| Parietal | 0.967 (0.045) | 0.994 (0.051) | **2.264 (0.024)** |
| Frontal | 0.935 (0.036) | 0.923 (0.029) | 1.689 (0.091) |
| Insula | 1.122 (0.077) | 1.137 (0.072) | 1.076 (0.282) |
| Subcortical | 1.260 (0.073) | 1.24 (0.064) | 0.957 (0.338) |
| Occipital | 0.902 (0.054) | 0.910 (0.046) | 0.552 (0.581) |
| Limbic | 1.060 (0.079) | 1.036 (0.046) | 1.397 (0.163) |
| Temporal | 1.014 (0.050) | 1.020 (0.032) | 0.473 (0.636) |
| Betweenness: |  |  |  |
| Parietal | 0.933 (0.178) | 1.036 (0.171) | **2.433 (0.015)** |
| Frontal | 0.877 (0.110) | 0.906 (0.080) | 1.599 (0.110) |
| Insula | 0.632 (0.179) | 0.648 (0.153) | 0.755 (0.451) |
| Subcortical | 2.343 (0.417) | 2.172 (0.366) | 1.532 (0.126) |
| Occipital | 0.855 (0.138) | 0.917 (0.107) | 1.892 (0.058) |
| Limbic | 1.406 (0.316) | 1.247 (0.188) | **2.174 (0.030)** |
| Temporal | 0.593 (0.111) | 0.563 (0.075) | 1.002 (0.316) |
| Local Efficiency: |  |  |  |
| Parietal | 0.930 (0.026) | 0.937 (0.029) | 1.239 (0.215) |
| Frontal | 0.997 (0.020) | 0.989 (0.022) | 0.969 (0.333) |
| Insula | 1.063 (0.044) | 1.070 (0.037) | 1.081 (0.280) |
| Subcortical | 0.946 (0.036) | 0.927 (0.035) | 1.903 (0.057) |
| Occipital | 1.002 (0.032) | 1.011 (0.031) | 0.743 (0.457) |
| Limbic | 1.032 (0.023) | 1.021 (0.032) | 1.183 (0.237) |
| Temporal | 1.053 (0.031) | 1.063 (0.021) | 1.746 (0.081) |

**Supplementary Figure 1.** Hubs distribution based on degree values in patients with AN and healthy women


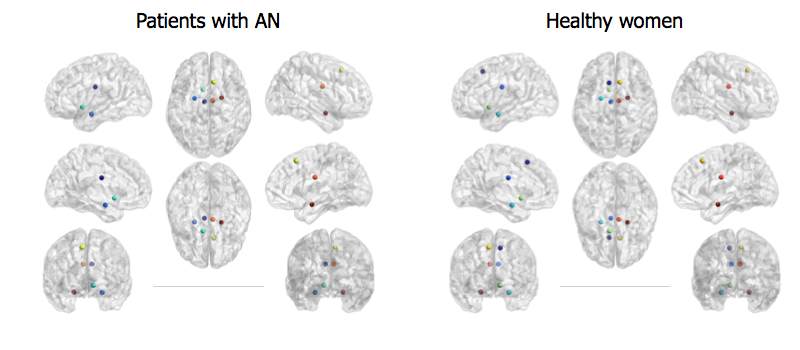

Supplement: Supplementary file 1 [file Table_1.DOCX]
